# Criteria for identification of *Schistosoma mansoni* eggs in faecal sediments prepared with the Helmintex method and stained by ninhydrin

**DOI:** 10.1590/0074-02760180529

**Published:** 2019-05-30

**Authors:** Renata Perotto de Souza, Vivian Favero, Vanessa Fey Pascoal, Catieli Lindholz, Hélio Radke Bittencourt, Carlos Graeff-Teixeira

**Affiliations:** 1 Pontifícia Universidade Católica do Rio Grande do Sul, Escola de Ciências, Porto Alegre, RS, Brasil

**Keywords:** schistosomiasis, egg-detection, ninhydrin, coproparasitology

## Abstract

Helmintex is a sensitive method used for detecting *Schistosoma mansoni* eggs. Here, we describe the observed frequency of six proposed criteria associated with the identification of *S. mansoni* eggs prepared with the Helmintex method and stained with ninhydrin. The efficacy of these criteria in classifying *S. mansoni* eggs when applied in various combinations was also examined. Nine observers registered the presence or absence of 6 different criteria in 100 eggs using a microscope at 100x magnification. Ninhydrin purple, which was frequently observed, was the criterion associated with the lowest inter-observer variability. At least three criteria were associated with a significantly better performance in egg identification. In conclusion, ninhydrin staining and a combination of criteria are recommended for microscope examination of faecal sediments.

An increasing number of countries have successfully implemented schistosomiasis control programs aimed at either eliminating this public health problem or interrupting its transmission.^1^ ^,^ ^2^ ^,^ ^3^ However, in areas with low infection intensity, classical diagnostic methods lack the sensitivity needed for diagnosing schistosomiasis.^4^ ^,^ ^5^ Helmintex (HTX) method is a procedure which has been recently developed for diagnosing schistosomiasis.^6^ This method uses the interaction between *Schistosoma* eggs and paramagnetic particles in a magnetic field to obtain a product that can then be stained using ninhydrin.^7^ However, despite the high sensitivity of this method, it involves a laborious process even after several modifications, such as new sieving procedures, inclusion of detergent in the fixation solution, and staining of eggs with ninhydrin for microscopic examination.^8^ Although the HTX method is not a “point-of-care” diagnostic method, it is considered as a reference method for detecting schistosomiasis due to its extremely high sensitivity.^9^

Favero et al.,^8^ proposed a set of criteria for the identification of *S. mansoni* (Sm) eggs in preparations obtained through the HTX method and stained with ninhydrin . These criteria include: 1) the presence of spines; 2) egg size: 150-117 µm x 70-40 µm;^10^ 3) egg shape; 4) definition of shell line; 5) internal space, and 6) colour. The objective of the current study was to evaluate these criteria by: (i) describing the frequency with which individual and combinatorial subsets of these six criteria occurred and (ii) evaluating the usefulness of these criteria for identifying *S. mansoni* eggs.

Filter papers containing positive faecal sediments were originally prepared as a part of a field study conducted in Candeal, Sergipe, in northeastern Brazil. The original study was approved by the Pontifícia Universidade Católica do Rio Grande do Sul (PUCRS) ethical committee (register 48809715.1.0000.5336) and the field and laboratory protocols used are described elsewhere.^9^ These filter papers were selected for re-examination in the present study.

The criteria for identifying *S. mansoni* eggs in HTX preparations stained with ninhydrin were proposed by experienced observers at the GPB-PUCRS laboratory. These six criteria were paired according to their contribution to a definitive diagnosis of Sm eggs (Fig. 1). The criteria were as follows: group A, criterion 1 ― presence of lateral spine; criterion 2 ― a well-defined shell outline; group B, criterion 1 ― ovoid shape; criterion 2 ― size; group C, criterion 1 ― space between shell and miracidium; criterion 2 ― purple colour. Different combinations of criteria from groups A, B, and C were also analysed in the following subsets: combination 1 ― the use of criteria from only 1 group; combination 2 ― the use of criteria from any two groups; and combination 3 ― having at least one criterion from all three groups.

**Fig. 1:**
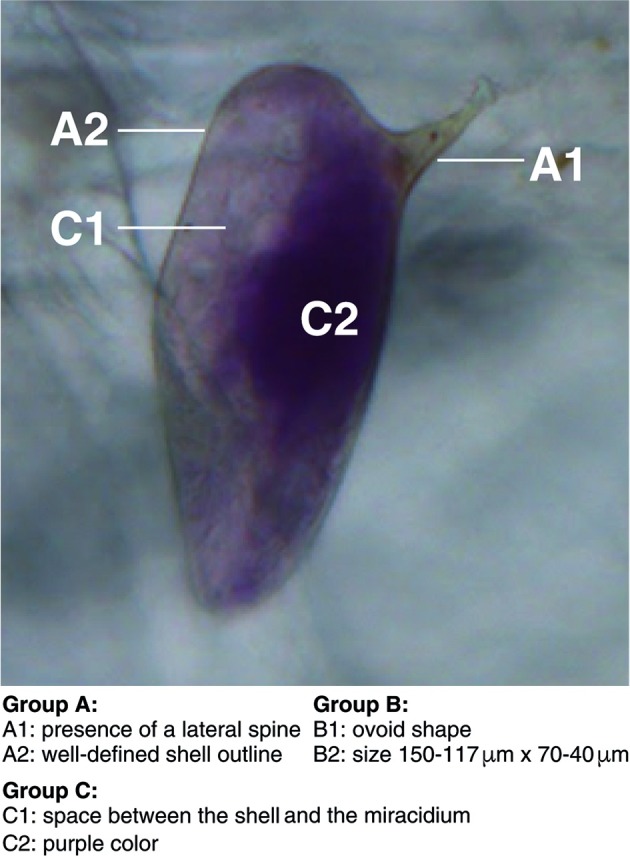
overview of the six observation criteria used in this study (divided into groups A-C) for the identification of *Schistosoma mansoni* eggs in sediments prepared according to the Helmintex method and stained with ninhydrin.

A group of nine readers simultaneously observed selected fields containing a total of 100 eggs on a flat screen monitor, and recorded their observations regarding the six possible criteria on a standardised form. All images shown were visualised with a ZEISS AXIO microscope equipped with a QIMAGE RESTINGA 7 camera and IMAGE PRO 7 software. The projected images were obtained at 100x magnification. Graphs were generated and statistical analyses were performed via IBM SPSS 20. Frequencies and confidence intervals (CI) for proportions were computed to compare the prevalence of criteria.

The proportional frequencies (the frequencies relative to the total number of observations) of each observed criterion ranged from 58% (shape) to 99% (purple colour) (Table). The criteria “ovoid shape” and “well-defined shell outline” (Fig. 1), had the highest inter-observer frequency variability, with 95%CI from 54.9% to 61.3% (range: 6.4) and 70.2% to 76% (range: 5.8), respectively. The best performance and highest inter-observer agreement were associated with combination 3 (observing at least one criterion from each group) (Fig. 2).

**Fig. 2:**
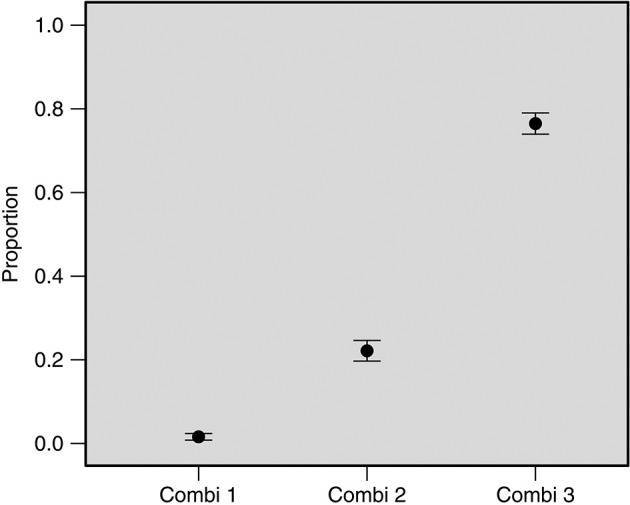
proportional frequency (95% confidence intervals) of combinations (Combi) one, two, and three criteria by nine observers. There were significant differences (p < 0.05) between proportional frequencies of all combinations.

The classification of criteria into groups A, B, and C was based on decreasing specificity for identifying *S. mansoni* eggs from other helminth eggs (Fig. 1). The presence of a lateral spine is a unique characteristic of *S. mansoni* eggs, and the use of this criterion for detecting *S. mansoni* eggs constitutes a highly specific diagnostic method. The criterion, “well-defined shell outline”, refers to a striking feature of many helminth eggs, such as eggs of *Ancylostoma* spp. and *Enterobius* spp., and the possibility that faecal sediment debris will present these features is very low. Undeformed eggs may present an appropriate size and/or shape for identification (e.g., group B criteria).

The purple colour produced by ninhydrin staining (group C criteria) is very helpful for quickly spotting eggs during initial microscopic screening of the filters. However, other debris may also be stained purple. While the purple colour criterion had the highest detection prevalence (99.7%) and the lowest inter-observer variability (the lowest distance between lower and higher confidence limits: 0.7), the use of this criterion alone, or any other criterion alone, was insufficient for egg identification. Moreover, a clear space between the miracidium and the eggshell (group C criteria) is not always present and is not specific for *S. mansoni* eggs. Therefore, except for the presence of a lateral spine and purple colour, accurate identification using the other four criteria requires more extensive observer training.

In areas with a high prevalence and intensity of infection, recognition of egg characteristics under a microscope is not a serious concern. However, in low endemic areas, more careful training and microscopic examinations are required, particularly where egg detection via microscopy is required as a reference method or cure control. Inter-observer variability may be improved by continuously and rigorously training observers and enhancing quality control measures. However, it was evident that combination 3 (observation of at least one criterion from each group) showed the best performance compared to combinations 1 and 2. There were significant differences (p < 0.05) between proportional frequencies of all combinations (Fig. 2).

**TABLE:** Six criteria for identification of *Schistosoma mansoni* eggs in Helmintex sediments stained by ninhydrin, their frequency, proportional frequency and limits of 95% confidence intervals (CI)

Criteria	n	Frequency	Proportion	95%CI	
				LL	HL
Well-defined shell outline	900	658	73,1%	70,2%	76,0%
Lateral Spine	900	693	77,0%	74,3%	79,7%
Size	900	794	88,2%	86,1%	90,3%
Shape	900	523	58,1%	54,9%	61,3%
Colour	900	897	99,7%	99,3%	100,0%
Space between shell and miracidium	900	683	75,9%	73,1%	78,7%

CI: confidence interval; n: number of observations; LL: CI lower limit; HL: CI higher limit.

In conclusion, the findings of the current study indicate that identification of *S. mansoni* eggs was successfully achieved using ninhydrin -stained HTX preparations, when at least one criterion from each group (A-C) was detected. Our findings also indicated that classification as “major/minor” criteria is not recommended. The proposed use of combinatorial observations for identifying *S. mansoni* eggs was found to be especially useful for egg specimens prepared according to the highly sensitive HTX method. Thus, the high sensitivity of the HTX method, combined with the use of multiple criteria for egg identification, may provide a more reliable diagnosis in settings characterised by extremely low infection intensities.
